# Development of lacrimal gland organoids from iPSC derived multizonal ocular cells

**DOI:** 10.3389/fcell.2022.1058846

**Published:** 2023-01-04

**Authors:** Melis Asal, Gamze Koçak, Vedat Sarı, Tuba Reçber, Emirhan Nemutlu, Canan Aslı Utine, Sinan Güven

**Affiliations:** ^1^ Izmir Biomedicine and Genome Center, Izmir, Turkey; ^2^ Izmir International Biomedicine and Genome Institute, Dokuz Eylül University, Izmir, Turkey; ^3^ Department of Analytical Chemistry, Faculty of Pharmacy, Hacettepe University, Ankara, Turkey; ^4^ Department of Ophthalmology, Dokuz Eylül University Hospital, Dokuz Eylül University, Izmir, Turkey; ^5^ Department of Medical Biology and Genetics, Faculty of Medicine, Dokuz Eylül University, Izmir, Turkey

**Keywords:** induced pluripotent stem cells, differentiation, developmental biology, stem cell, lacrimal gland, organoid

## Abstract

Lacrimal gland plays a vital role in maintaining the health and function of the ocular surface. Dysfunction of the gland leads to disruption of ocular surface homeostasis and can lead to severe outcomes. Approaches evolving through regenerative medicine have recently gained importance to restore the function of the gland. Using human induced pluripotent stem cells (iPSCs), we generated functional *in vitro* lacrimal gland organoids by adopting the multi zonal ocular differentiation approach. We differentiated human iPSCs and confirmed commitment to neuro ectodermal lineage. Then we identified emergence of mesenchymal and epithelial lacrimal gland progenitor cells by the third week of differentiation. Differentiated progenitors underwent branching morphogenesis in the following weeks, typical of lacrimal gland development. We were able to confirm the presence of lacrimal gland specific acinar, ductal, and myoepithelial cells and structures during weeks 4–7. Further on, we demonstrated the role of miR-205 in regulation of the lacrimal gland organoid development by monitoring miR-205 and FGF10 mRNA levels throughout the differentiation process. In addition, we assessed the functionality of the organoids using the *β*-Hexosaminidase assay, confirming the secretory function of lacrimal organoids. Finally, metabolomics analysis revealed a shift from amino acid metabolism to lipid metabolism in differentiated organoids. These functional, tear proteins secreting human lacrimal gland organoids harbor a great potential for the improvement of existing treatment options of lacrimal gland dysfunction and can serve as a platform to study human lacrimal gland development and morphogenesis.

## Introduction

Lacrimal gland is a tubuloacinar exocrine gland that secretes the aqueous layer of the tear film (Zoukhri, 2010; Örge and Boente, 2014; Garg and Zhang, 2017). Lacrimal gland mainly consists of acinar, ductal, and myoepithelial cells. Acinar cells make up the secretory units by synthesizing, storing and secreting water, electrolytes, proteins, and mucins in response to stimuli. Ductal cells form the gland ducts and modify the acinar cell secretion by secreting water and electrolytes (Bron et al., 2017). Myoepithelial cells surround acinar and ductal cells and apply pressure to acini to eject the secretion into the ducts and secrete the basal lamina. These cells are also thought to have a role in preserving the gland’s shape (Paulsen and Berry, 2006; Makarenkova and Dartt, 2015; Bron et al., 2017; Garg and Zhang, 2017). Dysfunction of the lacrimal gland leads to decreased tear production which results in poor ocular surface maintenance and makes it prone to infections (Conrady et al., 2016). Disturbance in aqueous tear secretion may cause dry eye disease with consequences as serious as loss of vision (Buckley, 2018). Current treatment options: artificial teardrops, anti-inflammatory drugs and punctal occlusion usually fail to prevent lacrimal gland atrophy, raising demand for alternative treatment options (Conrady et al., 2016).

In recent years, regenerative medicine approaches using stem cells have gained importance as a means of an alternative therapy option to restore the loss of lacrimal gland function (Zoukhri, 2010). Progress in tissue engineering and regenerative medicine approaches has been made in the development of the lacrimal gland in the last two decades. Mouse, rabbit, and human studies have been reported aiming to understand the gland physiology and maintain functionality of cultured embryonic and adult cells both *in vitro* and *in vivo* (Schrader et al., 2007; Ueda et al., 2009; Kobayashi et al., 2012; Tiwari et al., 2012; Hirayama et al., 2015; Lin et al., 2016; Gromova et al., 2017; Basova et al., 2020; Xiao and Zhang, 2020). Recent strategies focus on utilization of adult and pluripotent stem cells to unveil the regeneration capacity of lacrimal gland (Hirayama et al., 2017; Bannier-Hélaouët et al., 2021).

Organoids emerge as self-organized, multicellular, and functional miniature organs, with capacity to recapitulate healthy and diseased adult organ physiology. Induced pluripotent stem cells (iPSCs) possess the ability to differentiate into all three germ layers and make it possible to grow tissue specific organoids (Takahashi et al., 2007; Lancaster and Knoblich, 2014; Asal and Güven, 2020). This potential of human iPSCs emerges as a promising tool to develop a functional lacrimal gland organoid that can recover the loss of function in lacrimal gland disorders. So far, human iPSCs have been used to develop organoids of ocular lineage such as cornea, retina, and lens (Qiu et al., 2012; Erbani et al., 2016; Foster et al., 2017; Susaimanickam et al., 2017; Zhang et al., 2017; Chakrabarty et al., 2018; Hongisto et al., 2018). Recently Hayashi et al. demonstrated generation of lacrimal gland organoids from human iPSCs for the first-time. They generated self-formed ectodermal autonomous multi-zone (SEAM) of ocular cells by differentiating human iPS cells for 10–12 weeks. After that they sorted CD200−/SSEA-4+/ITGB4+ ocular surface epithelial stem cells and lacrimal-gland-like tissue organoids by further differentiating the cells for an addition of 20 days. Generated organoids showed similarity to native human lacrimal gland in terms of their morphology, as well as marker expression in the gene and protein levels (Hayashi et al., 2022).

Tissue targeted differentiation of iPSCs is achieved through recapitulation of specific developmental gene expressions, signaling and protein expressions. Different cell states demand specific metabolite levels to sustain their specialized functions. In many cases, the shift of metabolic plasticity from anabolic processes to catabolic processes or *vice versa* is a necessary step for cell fate determination (Folmes et al., 2012; Zeki et al., 2020). Comprehensive metabolite profiling, also known as metabolomics, determines the chemical phenotype of living organisms, disease incidence, severity, and progression. On top of that, it offers understanding of metabolic transformation triggers during pluripotent induction and differentiation (Folmes et al., 2011; Newgard, 2017).

In this study, we demonstrate *in vitro* generation of functional lacrimal gland organoids by using the differentiation potential of human iPSCs and following the multi zonal differentiation approach. We further show derivation of lacrimal tissue specific cells and reveal metabolomic profiling of the functional lacrimal gland organoids.

## Results and discussion

### Multi zonal differentiation gives rise to lacrimal gland progenitor cells

Human iPSCs were maintained in feeder-free conditions as colonies with defined borders typical for pluripotent cells (Supplementary Figure S1A). Pluripotency of cells was confirmed through TRA-1-60 and SSEA4 expression by flow cytometry (Supplementary Figure S1B) and OCT3/4, NANOG and SOX2 (Supplementary Figures S1C-D) by immunostaining.

**FIGURE 1:**
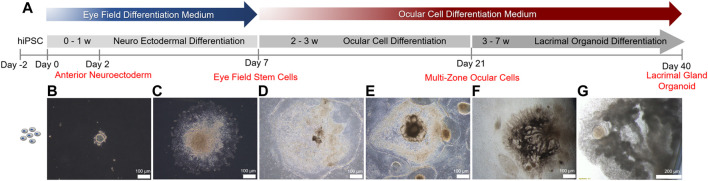
Timeline of lacrimal gland organoid differentiation from human iPSCs.

To recapitulate human ocular development, multi zonal differentiation approach was adopted (Li et al., 2019) (Figure 1A). Human iPSCs seeded as cell clumps within a Matrigel sandwich matrix (Figure 1B) committed to the neuro ectodermal lineage and spread to form cellular zones by day 7 (Figure 1C). Various identifiable zones comprised of different cell types with various morphologies and visible boundaries were formed by day 14 (Figure 1D). Five distinct zones with different cell morphologies, all committed to the ocular lineage, were present by day 21 (Figure 1E). These zones comprise of neural retina, retina pigment epithelium, ocular surface ectoderm, neural crest and lens, out of which the ocular surface ectoderm and neural crest are essential for lacrimal gland development (Li et al., 2019). Emergence of zone 3, the lacrimal gland epithelium progenitor ocular surface ectoderm, was confirmed by staining for the markers P63 and PAX6 (Supplementary Figure S2). Over the 7 weeks-long differentiation period, several multi-zonal colonies underwent elongation and branching, forming lacrimal gland ducts (Figure 1F). Branching was followed by budding and acinar organization typical of lacrimal gland development (Figure 1G).

**FIGURE 2:**
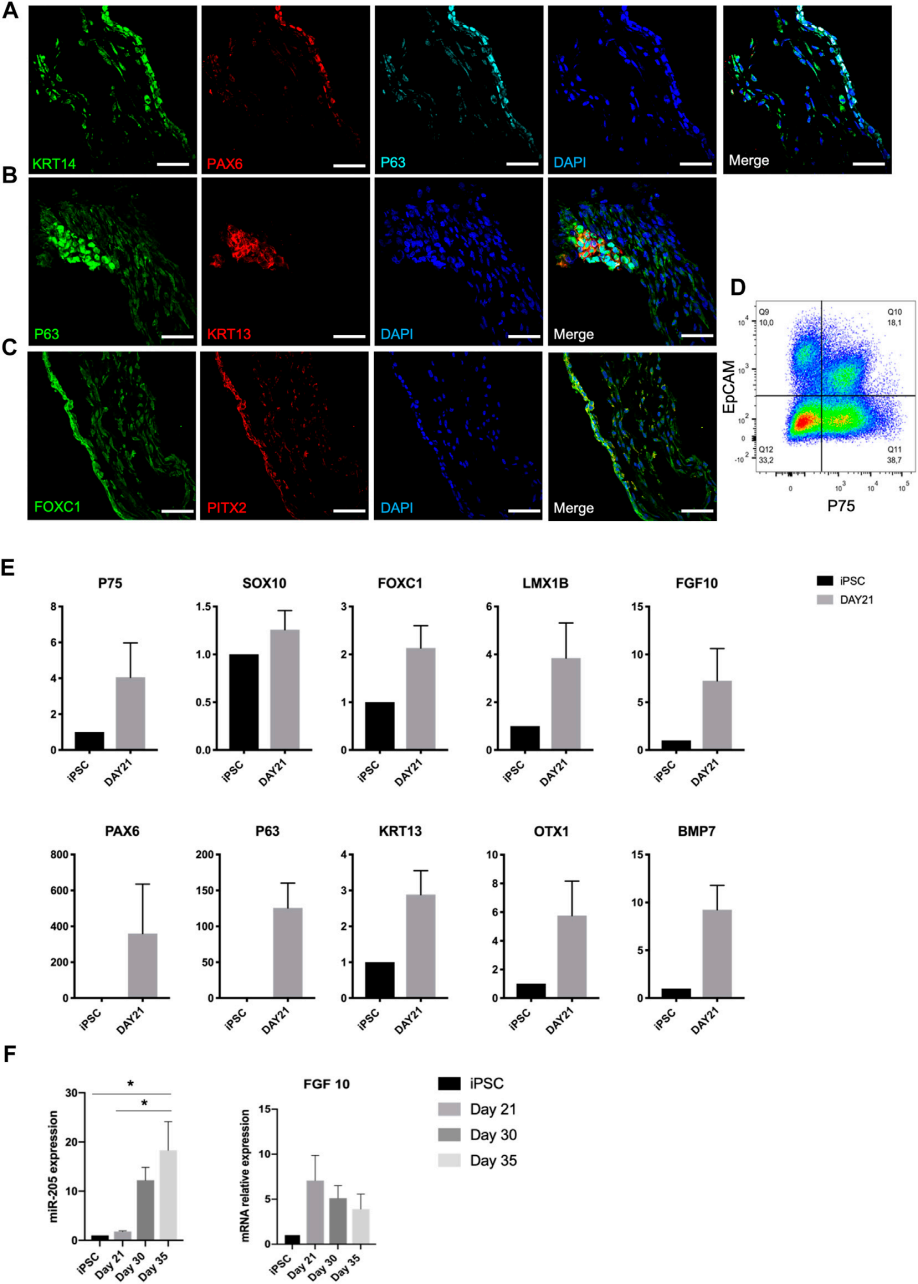
Expression of early lacrimal gland development markers in the protein and mRNA level on day 21 of differentiation confirms emergence of lacrimal gland stem and progenitor cells, **(A)** KRT14+/PAX6+/P63 + lacrimal gland multipotent epithelial stem cells, **(B)** KRT13+/P63 + lacrimal gland progenitor cells, **(C)** FOXC1+/PITX2+ periocular mesenchymal cells (Figures are representative of three independent differentiation experiments, scale bar: 50 µm), **(D)** Flow cytometry analysis of organoids with epithelial (EpCAM) and neural crest (P75) markers. N = 2 biological replicates. **(E)** Gene expression profile of organoids **(F)** miRNA 205 PCR expression and FGF10 levels during the same time course. (One-Way ANOVA. Error bars represent standard error of mean. *p ≤ .05, n = 3 biological replicates).

Lacrimal gland development requires epithelial-mesenchymal interaction (Hirayama et al., 2015). On Figure 2A, overlapping expression of KRT14, P63, and PAX6 depicts the ocular surface ectodermal cells, which are multipotent epithelial stem cells of lacrimal gland, on day 21 (Makarenkova and Dartt, 2015; Hirayama et al., 2016). These cells have the potential to give rise to KRT13 and P63 expressing conjunctival epithelial cells, which are the lacrimal gland progenitors (Basova et al., 2020). Presence of these progenitors was further confirmed by P63 and KRT13 immunostaining on day 21 (Figure 2B). Along with the emergence of epithelial cells, progenitor markers PAX6, KRT13 and P63 were identified in RNA level. Also, OTX1, whose absence leads to failure of lacrimal gland development in mice, was expressed in the RNA level (Figure 2E) (Garg and Zhang, 2017).

Periocular mesenchymal cells (FOXC1+, LMX1B+) are derived from neural crest (P75+, SOX10+) and produce FGF10, which has been identified as the main lacrimal gland inducer (Garg et al., 2017). Cells committed to the ocular cell lineage expressed the neural crest markers P75 and SOX10, and periocular mesenchyme markers FOXC1 and LMX1B by day 21 (Figure 2E). Emergence of periocular mesenchymal cells was confirmed with FOXC1 and PITX2 staining (Figure 2C). Co-presence of EpCAM + epithelial and P75 + mesenchymal (neural crest) cell populations was confirmed through flow cytometry analysis (Figure 2D), suggesting the potency of establishing the epithelial-mesenchymal interaction within developing organoids.

It was previously shown that FGF10 and BMP7 are inducers for bud formation and branching in mouse lacrimal gland (Makarenkova et al., 2000; Dean et al., 2004). FGF10 expression in mRNA level, most likely by developing periocular mesenchymal cells, was detected at the same time point (Figure 2E). In 2017, Farmer and others identified miR-205 as a regulator of mouse lacrimal gland development during the early stages of development. They suggested a role of miR-205 in repressing the targets that interfere with FGF10 signaling during lacrimal gland initiation (Farmer et al., 2017). Our findings support this reverse correlation also between miR-205 levels and FGF10 in human cell derived lacrimal gland organoids. A steady increase of miR-205 expression (Figure 2F) in relation to a decreasing trend in FGF10 mRNA expression (Figure 2F) was observed, suggesting the same role of miR-205 in human lacrimal gland development. BMP7 is expressed by periocular mesenchymal cells in the earlier stages of lacrimal gland development and both by the epithelium and mesenchyme in the latter stages. It is suggested to act on periocular mesenchymal condensation and proliferation (Dean et al., 2004). BMP7 expression of developing lacrimal gland organoids in mRNA level correlates with the emergence of periocular mesenchymal cells and is in line with earlier reports (Figure 2E).

### Lacrimal gland organoids consist of tissue specific acinar, ductal and myoepithelial cells

**FIGURE 3:**
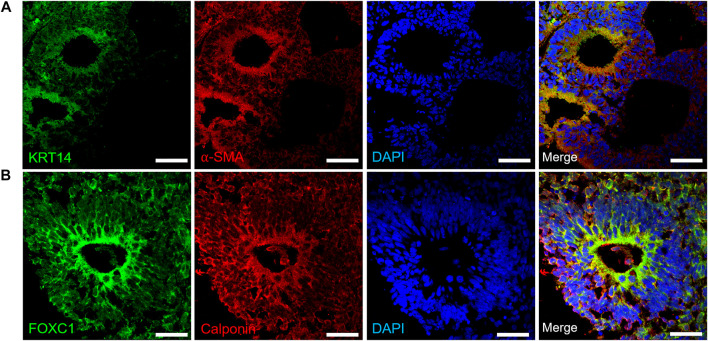
Expression of early lacrimal gland myoepithelial markers in the protein level **(A)** KRT14+/αSMA + lacrimal gland myoepithelial cells on day 30 **(B)** FOXC1+/Calponin + lacrimal gland myoepithelial cells on day 35 (Figures are representative of three independent differentiation experiments, scale bar: 40 μm).

Upon confirming the emergence of progenitor cells, we differentiated organoids (day 30–35) further towards lacrimal gland related cell phenotypes. Myoepithelial cells are positive for αSMA, and consecutively express KRT14, FOXC1 and Calponin (Basova et al., 2020). Kuony and Michon, 2017 reported that myoepithelial cells were KRT14+/αSMA+ in the mature mice lacrimal gland and demonstrated the similarity of lacrimal gland morphogenesis with other glands (Kuony and Michon, 2017). In our lacrimal gland organoids, expression of KRT14 and αSMA was found to be localized to the apical compartments of the organoids (Figure 3A). Similarly, FOXC1 and Calponin expression was localized to the apical regions (Figure 3B) of the cystic structures.

**FIGURE 4:**
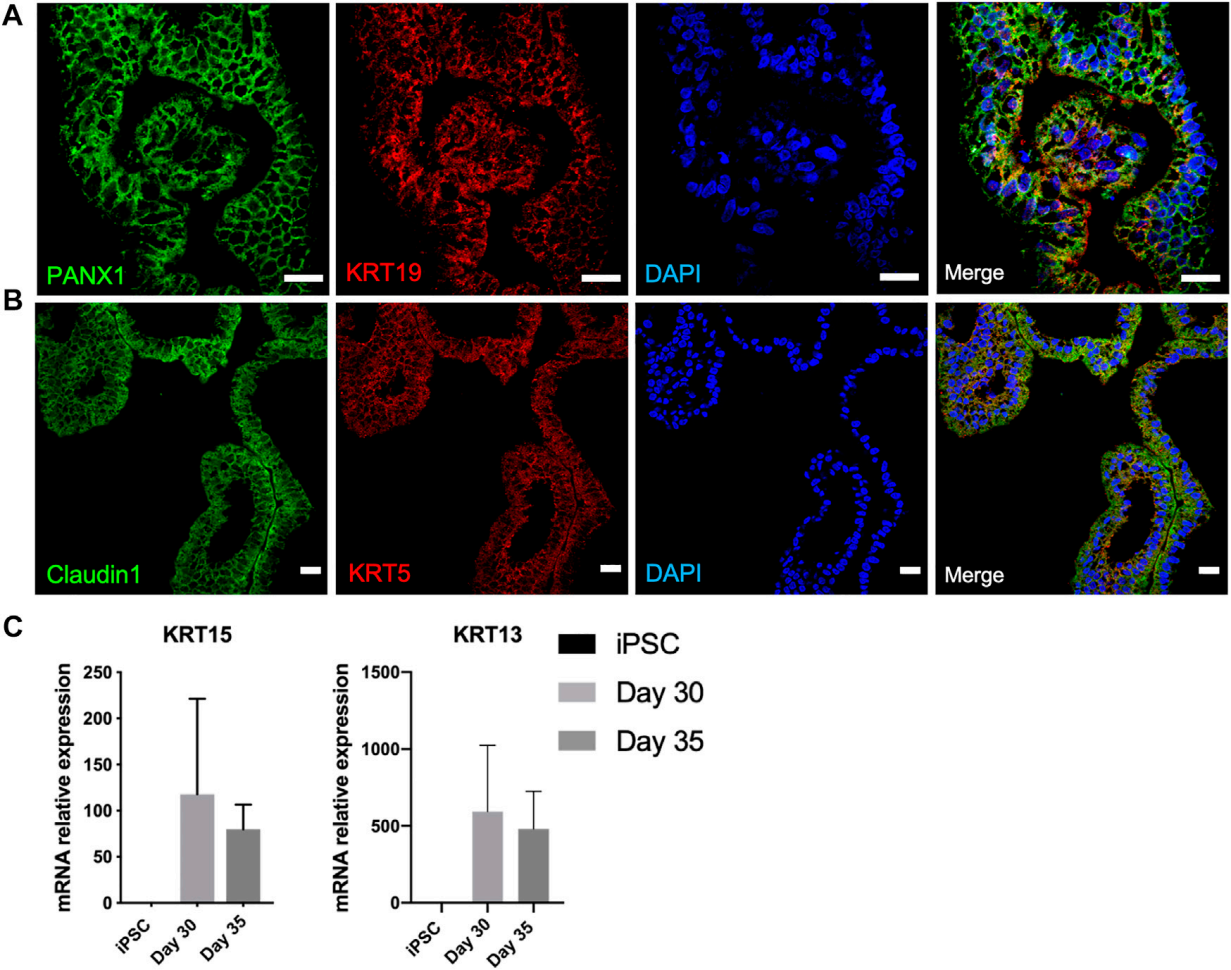
Characterization of developing lacrimal gland acinar and ductal cells on day 40 of differentiation **(A)** IF staining of PANX1+/KRT19 + lacrimal gland luminal ductal cells **(B)** IF staining of Claudin1+/KRT5+ lacrimal gland acinar cells (Figures are representative of three independent differentiation experiments, scale bar: 20 µm) **(C)** Decreasing trend in mRNA expression of lacrimal gland progenitor markers KRT15 and KRT13.

Organoids underwent branching morphogenesis after a month of differentiation (Figure 1). We hypothesized the branched structures to compound the luminal ductal and acinar cell subtypes of secretory units of functional lacrimal glands. Luminal ductal cells are characterized by PANX1 and KRT19. Immunostaining confirmed presence of these cells on day 40 of differentiation (Figure 4A). Acinar/ductal cells of the lacrimal gland and are marked by Claudin1 and KRT5 expression (Basova et al., 2020). These cells have been detected as forming cystic structures on day 40 of differentiation (Figure 4B).

KRT15 is expressed by the lacrimal gland progenitor cells, thus referred to as specific marker of the developing epithelium (Hirayama et al., 2015). Expression of KRT15 in mRNA level was detected on day 30 and was further maintained on day 35 (Figure 4C). This finding can be correlated to earlier findings (Makarenkova and Dartt, 2015), as the lacrimal gland formation is achieved by day 35 of differentiation, supported by the emergence of luminal ductal and acinar cells. Likewise, mRNA levels of KRT13, which is expressed by lacrimal gland progenitors decreased by day 35, suggesting a later stage of lacrimal development (Figure 4C).

### iPSC derived lacrimal gland organoids secrete aqueous tear proteins

**FIGURE 5:**
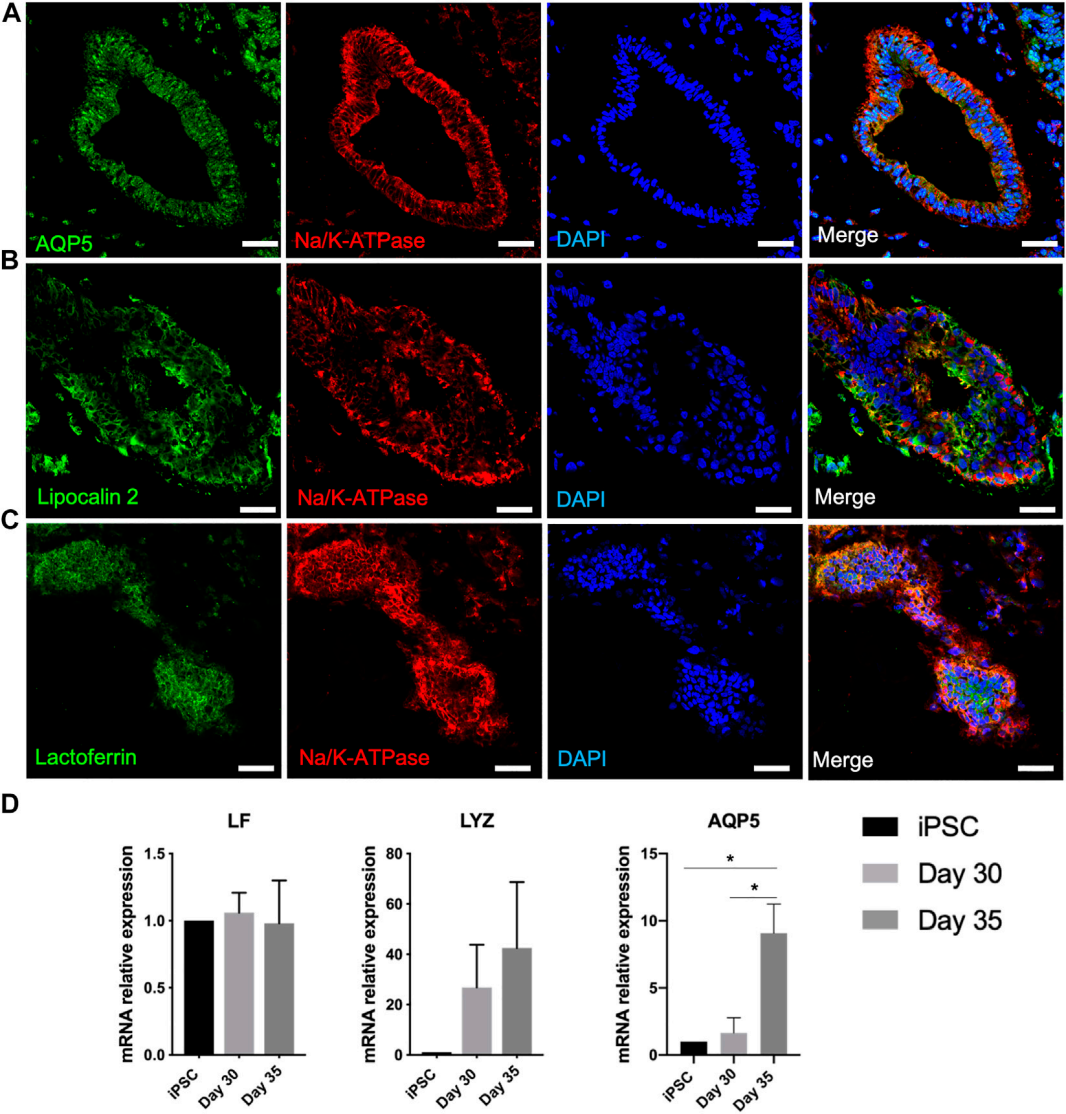
Expression of lacrimal gland maturity markers in the protein and mRNA level on day 40 of differentiation confirms maturation of lacrimal gland structures **(A–C)** Acinar cells (Figures are representative of three independent differentiation experiments, scale bar: 40 µm) **(D)** Expression of markers related to secretory function of lacrimal gland organoids (One-Way ANOVA. Error bars represent standard error of mean. *p ≤ .05, n = 3 biological replicates).

Lacrimal gland acinar cells secrete water, electrolytes, tear proteins and mucins (Bron et al., 2017). AQP5, a water channel protein and Na+/K + ATPase, an ion exchange enzyme expressed by lacrimal gland acinar cells are suggested to have active roles in tear secretion. After 6-7-week culture of developed organoids, branched structures appeared to have further differentiated into lacrimal gland tissues. AQP5 appeared to be localized around the cystic structures, along with basolateral expression of Na+/K + ATPase, supporting the development of lacrimal gland acini formation (Figures 5A–C) (Ding et al., 2011). Significantly elevated expression of AQP5 by day 35 in the mRNA level also suggests maturated lacrimal gland organoids (Figure 5D).

Lysozyme, lactoferrin, and tear lipocalins are key tear proteins (Glasgow, 1995). Lysozyme, a major component of the lacrimal gland secretion, was detected in the mRNA level on day 30 of differentiation, with an increased expression by day 35 (Figure 5D). Lactoferrin expression was not found to be increased significantly in the mRNA level (Figure 5D). This might be due to lack of innervation, which most likely led to insufficient/absent stimulation of secretion. Tear lipocalin, an iron-sequestering protein, is a biomarker for a variety of diseases, including dry eye disease (Dartt, 2011). Lipocalin was expressed by the developed lacrimal gland organoids (Figure 5B).

**FIGURE 6:**
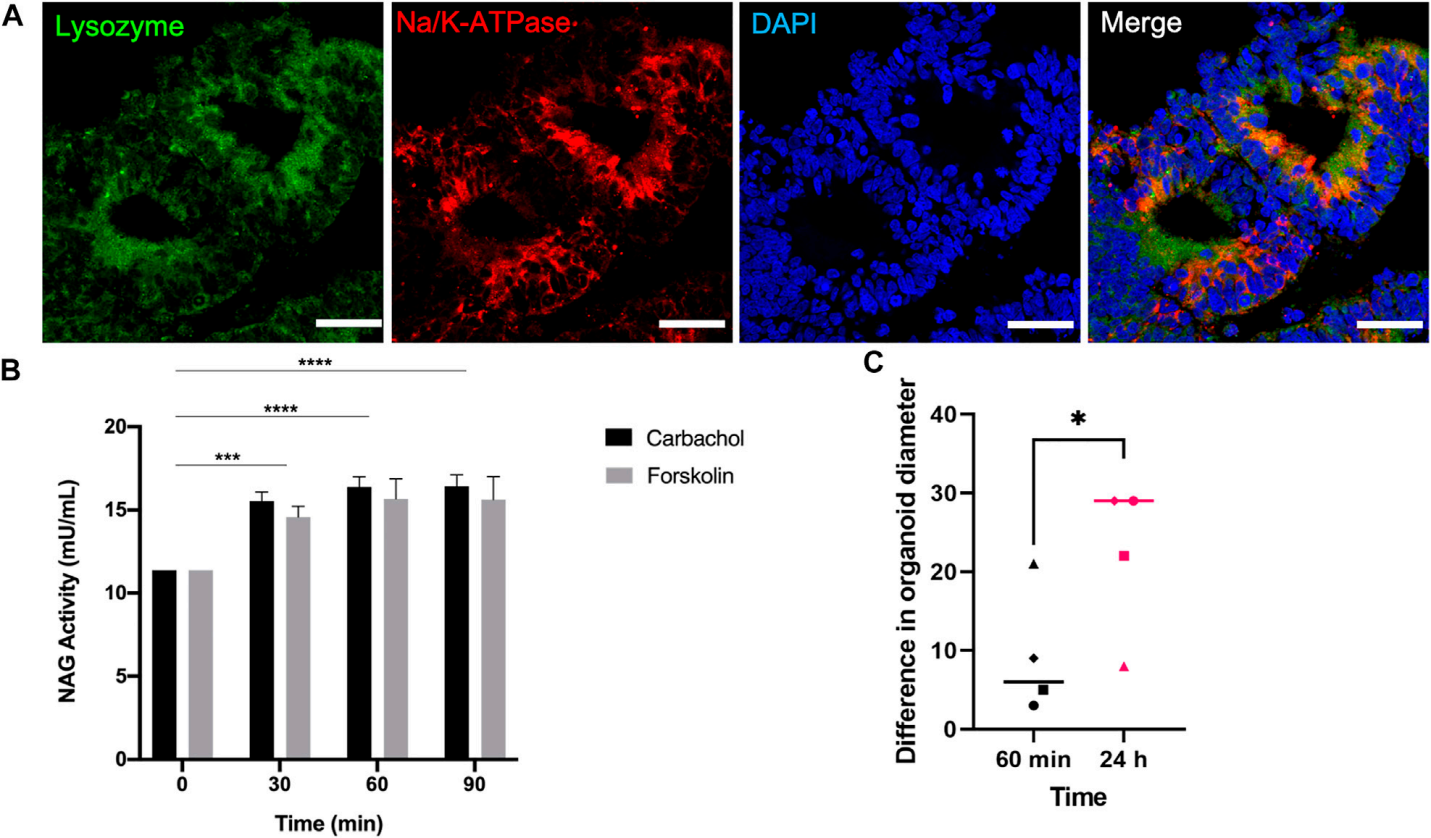
Expression of the functionality marker lysozyme and secretory levels of the active lysozyme enzyme NAG on day 40 of differentiation **(A)** IF staining of Lysozyme+/Na+/K + ATPase lacrimal gland acinar cells (Figures are representative of three independent differentiation experiments, scale bar: 40 µm) **(B)** NAG concentration in the supernatant of lacrimal gland organoids following carbachol and forskolin stimulation (Two-Way ANOVA. Error bars represent standard error of mean. ***p ≤ .001; ****p ≤ .0001, n = 3 biological replicates). **(C)** Swelling of organoids following carbachol and forskolin stimulation (Unpaired *t*-test. Error bars represent standard error of mean. *p ≤ .05, n = 4 biological replicates).

Lysozyme, which was detected in the mRNA level during day 30 and 35 of differentiation (Figure 5D), was expressed in the protein level on day 40 along with Na+/K + ATPase around the cystic structures (Figure 6A). This finding further confirms the presence of acinar cells and suggests a secretory function of the lacrimal gland organoids.

To further investigate the secretory function, organoids were stimulated with carbachol and forskolin. β-Hexosaminidase assay was employed to measure the concentration NAG, which is the active lysozyme enzyme found in tears (Lu et al., 2017; Jeong et al., 2021). Treating the organoids with either stimulant for 30, 60, and 90 min led to significant levels of NAG (Figure 6B), confirming the lysozyme secretion ability of the developed organoids. NAG concentration has been used to assess secretory ability of lacrimal gland spheroids in the literature previously (Lu et al., 2017). Lu and others grew primary rabbit lacrimal gland organoids. They stimulated the organoids with 100 μM carbachol for 30 min and detected .2 mU/mg of NAG in the supernatant in 3D (Lu et al., 2017). Likewise, Jeong and others used β-Hexosaminidase assay to assess the secretory function of primary human lacrimal gland cultures after stimulating with pilocarpine (Jeong et al., 2021). They observed an increase in Ca^2+^ and NAG secretion. Also, treating organoids for 24 h led to significant increase in organoid diameter compared to 60-min stimulation (Figure 6C).

### Stimulated lacrimal gland organoids show a metabolite profile shift

**FIGURE 7:**
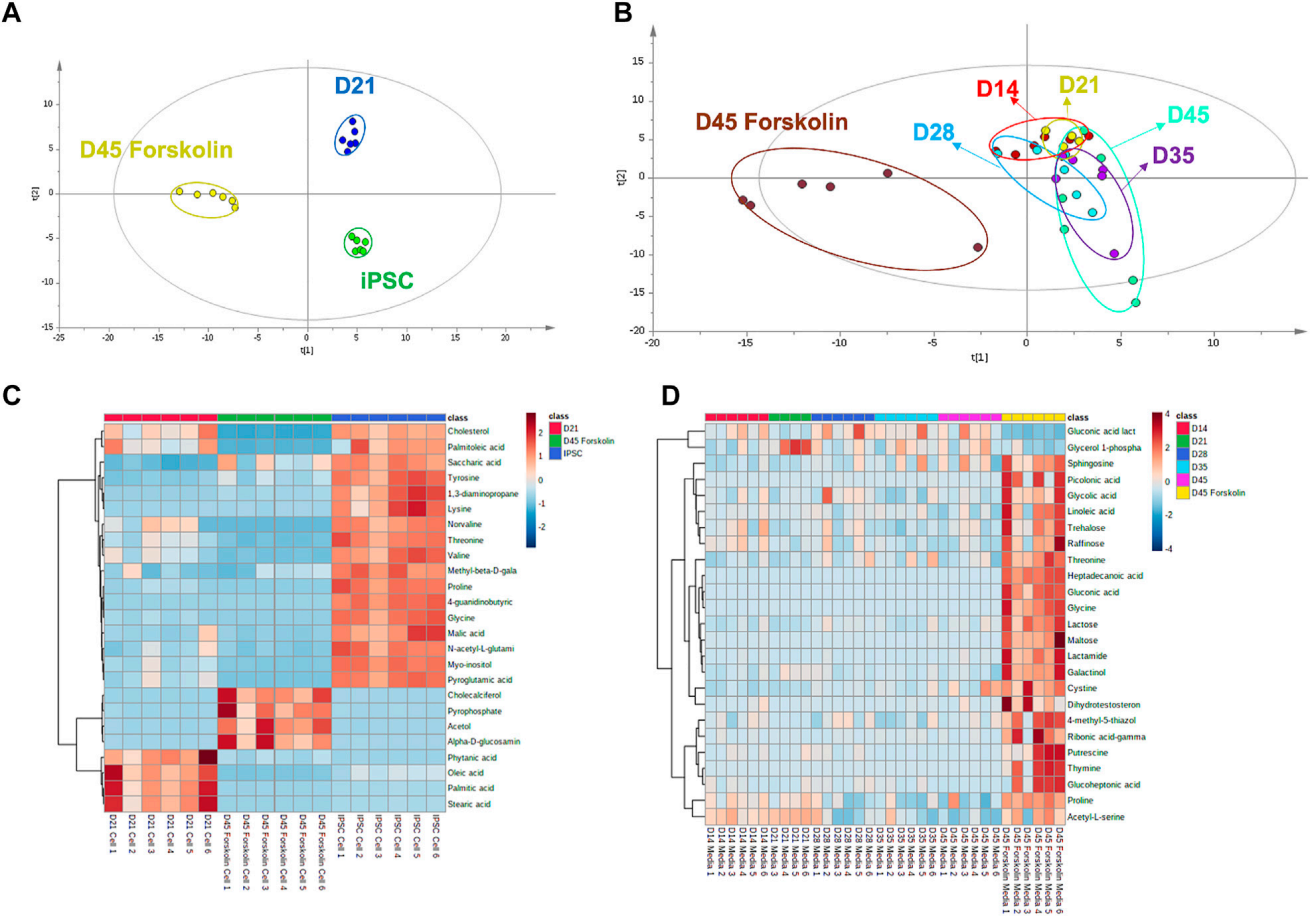
Metabolomic analysis of cell and culture medium **(A)** PLS-DA score plot for metabolomic profiles of cell lines at different time points and presence of the stimulant (*R* ^2^ = .820 and Q^2^: .978) **(B)** PLS-DA score plot for exometabolomic profiles of cell culture mediums (*R* ^2^ = .410 and Q^2^: .117) **(C)** Heat map for cell metabolites **(D)** Heat map for cell culture medium metabolites (n = 6 biological replicates).

A GC–MS-based metabolomic profiling study was performed in iPSC derived lacrimal gland cells for fingerprint analysis and media samples for footprint analysis (Supplementary Figure S3). 127 and 178 metabolites respectively in cell and culture media samples were identified (Supplementary materials). Metabolomic profiles were visualized with multivariate statistical analysis using partial least square discriminant analysis (PLS-DA), and heatmap (Figure 7). The statistical goodness and robustness of the PLS-DA models were evaluated using R^2^ (the fraction of variance explained by a component) and Q^2^ (the fraction of the total variation predicted by a component) respectively. High R^2^ and Q^2^ values indicate the robustness of the methods.

Metabolites from cells at pluripotent state, differentiated state at day 21 and forskolin stimulated state at day 45 showed distinct clustering (Figure 7A). Clear separation of metabolic profiling without any overlap during the differentiation of iPSCs confirm the differentiation in the metabolomics level. During the organoid derivation, three different cell culture media were used. These media were applied as follows: pluripotent cell medium for iPSC culture, initial eye field cell differentiation medium (EM) for first 7 days, ocular cell differentiation medium (OD) for further differentiation and maturation. Exometabolomics analysis revealed a clear separation based on the medium type (Supplementary Figure S3A). When the culture period in OD medium was further analyzed, a clear shift in metabolite clusters indicating the differentiation and maturation of organoids (Figure 7B) was revealed. Moreover, a noticeable metabolite profile shift upon the stimulation with forskolin compared to unstimulated controls was observed.

A drastic shift from amino acid metabolism to lipid metabolism was observed when metabolomic profile of iPSCs were compared to that of lacrimal gland organoids at day 21 (Figure 7C). Higher lipid levels in the lacrimal gland indicated that the cells have acquired their lacrimal phenotype at the functional level. According to literature, taurine, L-glutamic acid, and L-glutamine are the most abundant, while L-valine, L-isoleucine, L-methionine, L-asparagine, tyrosine, L-histidine, and L-ornithine are the least abundant amino acids in healthy tear (Nakatsukasa et al., 2011). We observed a similar amino acid distribution, with low concentrations of L-tyrosine, L-valine, L-proline, and L-glycine in the lacrimal gland organoids. Once the lacrimal cells were stimulated with forskolin, levels of cholecalciferol, pyrophosphate, acetol and alpha glucosamine phosphate were elevated whereas the fatty acid and cholesterol levels decreased. Forskolin stimulation also led to an increase in glucoheptonic acid, heptadecanoic acid, gluconic acid, glycine, glycolic acid, and threonine levels (Figures 7C,D), likely due to high energy requirements in the presence of the stimulant.

## Conclusion and future aspects

Here we report using human iPSCs to obtain all lacrimal gland major cell types: acinar, ductal and myoepithelial, by adopting the multi zonal differentiation approach. Previous studies performed with adult lacrimal gland stem/progenitor cells showed acinar or ductal differentiation with different culture methods and stiffness of matrices (Gromova et al., 2017; Xiao and Zhang, 2020). Adult lacrimal gland stem cells’ ability to form functional organoids has been a breakthrough as artificial tear drops, the most common treatment to restore the loss of tears, lack proteins and peptides that are essential for ocular surface homeostasis (Zoukhri, 2010; Örge and Boente, 2014; Garg and Zhang, 2017). However primary human lacrimal tissue is scarce and does not enable patient specific applications such as personalized medicine.

Utilization of iPSCs to generate lacrimal gland organoids as a tool for aqueous tear secretion improvement and treatment stands out as a more comprehensive option. Previously, in a very promising study Hirayama and others differentiated human embryonic stem cells (hESC) into lacrimal gland epithelial cells with the overexpression of the transcription factors involved in the organogenesis of mice lacrimal gland. However the functionality of these cells is not assessed in terms of secretory capability, and the maturity of the cells is not well addressed. In addition to this, use of mice lacrimal gland developmental transcription factors can cause discrepancies, and the use of hESCs might cause ethical problems (Hirayama et al., 2017). More recently, Hayashi and others have shown that human iPSC derived lacrimal gland organoids undergo branching morphogenesis under well identified *in vitro* culture conditions. In addition to this, the organoids are shown to have striking similarities to the native lacrimal gland. However, the method employed in this study is complex and lengthy (10–17 weeks in total) compared to our approach which gives rise to functional cells in as short as 7 weeks (Hayashi et al., 2022).

In this study, iPSC-derived lacrimal gland organoids’ secretion ability was not only assessed with cell type related markers but also with lysosomal secretion analysis following forskolin and carbachol stimulation. These results show that our organoid model is capable of secreting tear components. However, NAG activity is measured in the medium, from the basal side. Considering that tears are secreted apically, towards the lumen of the organoids, it would be interesting in the future to sample the lumen of the organoids and measure the NAG activity directly. Nevertheless, the human iPSC derived organoids offer a great potential for *in vitro* tear production.

Farmer and others identified the non-coding RNA, miR-205, as a critical regulator through *Fgf10* for mice lacrimal gland embryonic development (Farmer et al., 2017). Our study showed a correlation between miR-205 and FGF10 expression throughout human iPSC derived lacrimal gland organoid differentiation, suggesting similar developmental processes. The regulatory role of miR-205 in human lacrimal gland development can be further investigated by overexpressing and knocking down miR-205 in the organoids.

Metabolomic profiling of developed lacrimal gland organoids also confirms the differentiation in the level of metabolomics. Further integration of omics technologies to the lacrimal gland organoids will potentially enable to advance our knowledge in lacrimal gland associated diseases and to designate candidate therapeutics and biomarkers.

The presented iPSC-derived lacrimal gland organoid model also holds great potential to study lacrimal gland development and morphogenesis. In the future the developed organoid model can be improved to a greater extent by removing the other ocular cell types that coemerge. Recently, Hayashi et al., 2022 showed how to physically select lacrimal gland progenitor cells from multi zonal heterogenous populations depending on the phenotype. Similar approaches should be employed to strategy presented in this study to demonstrate improved budding and branching morphogenesis of lacrimal gland organoids.

Furthermore, by using patient derived iPSCs, development of genetic lacrimal gland diseases such as aplasia of the lacrimal and salivary glands and lacrimo-auriculo-dento-digital syndrome can be studied.

The organoids can as well offer a platform for the study of dry eye disease and testing of candidate drugs. It was shown that oxidative stress has a role in the development of Sjögren syndrome, which causes severe DED (Pellegrini et al., 2020). An interplay between oxidative stress and inflammation in the lacrimal gland was also revealed (de Souza et al., 2021). Therefore, nutritional medication such as polyphenols that target the antioxidant pathway Nrf2, and anti-inflammatory lipid mediators such as lipoxin A4 can be employed to induce lacrimal gland protection (Scuto et al., 2019; Scuto et al., 2021; Scuto et al., 2022). The organoids can be treated with these components for the assessment of efficacy.

Additionally, lacrimal gland organoids can be integrated with organ-on-chip platforms to allow shape-guided morphogenesis during development, to study the mechanics and the physiological response of the organoids, and their interaction with other organs (Nikolaev et al., 2020).

## Materials and methods

### hiPS cell culture

hiPS cells generated by Akbari et al., 2019 were used in this study (Akbari et al., 2019). Three healthy hiPSC lines were evaluated for their lacrimal gland differentiation efficiency. All three cell lines were able to give rise to lacrimal gland organoids. Non-etheless, one cell line was chosen for this study due to its fast growing capacity. hiPSCs were maintained under feeder-free culture conditions on six well plates coated with hESC qualified Matrigel (Corning), with mTeSR1 medium (STEMCELL Technologies). Media was changed daily and hiPSCs were passaged every 6–7 days at a ratio of up to 1:10 using ReleSR (STEMCELL Technologies).

### Lacrimal gland organoids from hiPS cells

Multi zonal ocular cell differentiation approach developed by Li and others was adopted for lacrimal gland differentiation (Li et al., 2019). hiPS cells were seeded on 1% hESC qualified Matrigel-coated six well plates and maintained in eye field differentiation (ED) medium composed of (DMEM/F12 (Gibco, Thermo Fisher Scientific) and neurobasal medium (Gibco, Thermo Fisher Scientific) (1:1) supplemented with 2 mM L-GlutaMAX (Gibco, Thermo Fisher Scientific), 0.1 mM non-essential amino acids (Lonza), 0.1 mM monothioglycerol (FUJIFILM Wako Pure Chemical Corporation), and 1% N2 MAX supplement (R&D Systems). 2% Matrigel was added to ED medium for first 2 days of differentiation. After 7 days of culture, medium was replaced with the ocular cell differentiation (OD) medium composed of DMEM/F12 supplemented with 10% knockout serum replacement (Gibco, Thermo Fisher Scientific), 2 mM L-GlutaMAX, 0.1 mM NEAA, and 0.1 mM monothioglycerol. Organoids maintained in culture for up to 45 days with daily medium refreshments.

### Immunofluorescence staining

iPSC-derived cells were fixed with 4% paraformaldehyde (Sigma-Aldrich) for 20 min at room temperature and embedded in cryomatrix (OCT, Fisher Healthcare). 5 μm thick serial sections were blocked/permeabilized with staining solution (1% Bovine Serum Albumin (BSA; Sigma-Aldrich) (w/v) and .3% Triton-X100 (neoFroxx) (v/v) in PBS) for 1 h at room temperature. Cells were then treated with primary antibodies at 4°C overnight and afterwards with secondary antibodies for 1 h at room temperature. Nuclei were stained with 4,6-diamidino-2-phenylindole (.5 μg/ml) (DAPI; Neofroxx). iPSCs were treated with the staining solution for blocking and permeabilization after fixation and staining proceeded as previously explained. Samples were visualized either with Fluorescence Microscopy (Olympus IX71) or Confocal Microscopy (Zeiss LSM880). Antibodies used are listed in Supplementary Table 1.

### RNA isolation and RT-qPCR

Total RNA extraction was performed with TRIzol reagent, and samples were stored at −80°C until processed. RNA was isolated with Nucleospin RNA isolation kit (Macherey-Nagel) according to the manufacturer’s instructions. cDNA was synthesized using OneScript Plus cDNA Synthesis Kit (Applied Biological Materials). RT-PCR was carried out using GoTaq Master Mix (Promega). qPCR was performed on the Roche LightCycler 480 instrument for 2 min at 95°C followed by 40 cycles (95°C for 10 s, 60°C for 10 s and 72°C for 30 s). A melting step (95°C for 10 s and 65°C for 30 s) and a cooling step (40°C for 30 s) were added. All PCR reactions were performed in triplicates. Gene expression levels were normalized to the endogenous control glyceraldehyde 3-phosphate dehydrogenase (GAPDH) and the fold change relative to the control was calculated using the 2^−Δ(ΔCt)^ method. Primers used for mRNA levels are listed in Supplementary Table 2.

### miRNA quantitative PCR

iPSC-derived lacrimal glands were treated with TRIzol reagent and miRNA was isolated using mirVANA miRNA isolation kit (Ambion/RNA by Life Technologies, United States) according to manufacturer’s instructions. Expression of miR-205 was quantified using TaqMan™ MicroRNA Assay kit (Thermo Fisher Scientific) using Roche LightCycler 480 Probes Master (Roche). miR-205 levels were normalized to RNU6B (Thermo Fisher Scientific) and the fold change relative to the control was calculated using the 2^−Δ (ΔCt)^ method.

### Flow cytometry

iPSCs and iPSC-derived cells were harvested with Accutase (Gibco, Thermo Fisher Scientific) and resuspended in ice-cold ocular differentiation medium. Cells were filtered through a 40 μm pore size cell strainer (Corning) and stained with Zombie UV Fixable Viability Kit (Biolegend) for 10 min on ice. After washing, samples were treated with antibodies on ice for 30 min and washed before acquisition on BD Fortessa. Results were analyzed using the FlowJo software (TreeStar, San Carlos, CA). Antibodies used for flow cytometry are depicted in Supplementary Table 3.

### β-hexosaminidase assay

Secretory function of lacrimal gland organoids was quantified by measuring N-acetyl-β-glucosaminidase (NAG), a lysosomal enzyme in the tear fluid. In brief, organoids were treated either with 10 µM forskolin (Tocris) or 100 µM carbachol (Merck) for 30, 60, and 90 min. Bright field images of the treated cultures were acquired at the initiation of treatment and after 30 min, 60 min and 24 h. Acquired images were quantified using the measure function of the ImageJ software to assess the organoid swelling. Treated culture medium and control medium for background was collected and centrifuged at 10,000 x g for 3 min. NAG concentration in supernatants was quantified using a NAG assay kit (Abcam, ab204705) following the manufacturer’s protocol. Reaction product was detected colorimetrically at 400 nm using a microplate reader (Multiskan GO, Thermo Fisher Scientific).

### Sample preparation for metabolomics analysis

Lacrimal gland organoids cultured for 21 days and 45 days (stimulated with forskolin for 5 min) were washed with .9% NaCl solution (Sigma-Aldrich) and fixed with methanol:water (9:1, v/v) (Sigma-Aldrich). Samples were frozen in liquid nitrogen and scraped into 2 ml Eppendorf tubes. Tubes were centrifuged at 15,000 rpm for 15 min and supernatants were transferred into 2 ml Eppendorf tubes and stored at −80°C until analysis. Culture media samples were collected on days 1, 7, 14, 21, 28, 35, and 45 and stored at −80°C.

### Metabolomic analysis by GC-MS

Samples stored at −80°C were thawed at room temperature and evaporated to complete dryness in a vacuum centrifuge (Savant ISS110 SpeedVac Concentrator Thermo Scientific). Dried samples were methoxylated with 20 µL of methoxyamine hydrochloride (20 mg/ml in pyridine) by incubating in an oven at 30°C for 90 min 80 µL of N-methyl-N-trimethylsilyl trifluoroacetamide (Sigma-Aldrich) and 1% trimethylchlorosilane (Sigma-Aldrich) were added and samples were further incubated at 37°C for 30 min. Derivatized samples were transferred into silylated GC-MS vials and analyzed by the GC-MS system (Shimadzu -QP2010 Ultra) using a DB-5MS stationary phase column (30 m + 10 m DuraGuard × .25 mm i. d. and .25-μm film thickness). The solvent delay was set for 5.90 min. Oven temperature was initially held at 60°C for 1 min. Afterwards, the temperature was raised with a gradient of 10°C/min until 325°C and held for 10 min before cool-down. The MSD transfer line temperature was set to 290°C. The flow through the column was held constant at 1 ml He/min. The mass range was 50–650 Da. The run time was 37.5 min.

Data deconvolution, peak alignment, normalization, and data matrix creation were carried out using the MS-DIAL (ver. 4.0) software. Metabolite identification for GC-MS was done using a commercially available retention index library (Fiehn Retention Index Library) with a 70% of higher identification cut-off score. The data matrix obtained from MS-DIAL was transferred to an Excel work file. Any metabolite traits having more than 50% of the values missing were excluded from the data matrix. Missing values in the data matrix were filled with the half value of the smallest concentration in the metabolite group. The final data matrix was imported into the SIMCA-P+ (v13.0, Umetrics, Sweden) and Metaboanalyst software for multivariate analyses. Within the scope of multivariate analyzes, principal component analysis (PCA), partial least squares differentiation analysis (PLS-DA), and heatmap analysis were performed.

### Image processing

Processing of the images was performed using Zen Blue and ImageJ softwares. Linear contrast and brightness transformations were applied in the same manner to all comparable images to clarify the data.

### Statistical analysis

All experiments were repeated three times. Data are represented as the mean ± SEM. Statistical analyses were performed using GraphPad Prism 8.0 (GraphPad Software, San Diego, United States). Differences between groups were considered significant at *p ≤ .05, ***p ≤ .001, ****p ≤ .0001).

## Data Availability

The original contributions presented in the study are included in the article/Supplementary Material, further inquiries can be directed to the corresponding author.
