# Supplementary material for: Development of lacrimal gland organoids from iPSC derived multizonal ocular cells
Source: Front Cell Dev Biol. 2023 Jan 4;10:1058846. doi: 10.3389/fcell.2022.1058846 (PMC9846036; doi:10.3389/fcell.2022.1058846)
Supplement: Supplementary file 1 [file Table1.DOCX]

**Supplementary Table 1. Antibodies used for immunofluorescence staining**

| **Target** | **Product number** |
| --- | --- |
| **OCT3/4** | R&D, MAB1759 |
| **NANOG** | Thermo Fisher, PA5-18406 |
| **SOX2** | Bioss, bs-5685R |
| **PITX2** | Novus, NBP2-57409 |
| **FOXC1** | Novus, NB100-1268 |
| **PAX6** | Novus, NBP2-44576 |
| **TP63** | R&D, AF1916 |
| **KRT13** | Novus, NBP2-38166 |
| **AQP5** | Bioss, bs-1554R |
| **Na^+^/K^+^ ATPase** | Millipore, 05-369-25ug |
| **KRT14** | Sigma, SAB5500124 |
| **SOX10** | Cell Signaling, 89356 |
| **a-SMA** | Cell Signaling, 48938S |
| **PANX1** | Sigma, HPA016930 |
| **KRT19** | Cell Signaling, 4558 |
| **KRT5** | Thermo, MA517057 |
| **Lysozyme** | Thermo, PA5-16668 |
| **Claudin1** | Cell Signaling, 13995S |
| **Lactoferrin** | ABCAM, ab109216 |
| **LCN 2** | ABCAM, ab41105 |
| **Calponin** | Bioss, bs-0095R |
| **Anti-rat-AF488** | Jackson Immuno, 712-545-150 |
| **Anti-goat-AF488** | Jackson Immuno, 705-545-003 |
| **Anti-goat-AF647** | Jackson Immuno, 705-605-003 |
| **Anti-rabbit-AF594** | Jackson Immuno, 711-585-152 |
| **Anti-rabbit-AF488** | Jackson Immuno, 711-545-152 |
| **Anti-mouse-AF594** | Jackson Immuno, 715-585-150 |
