# Supplementary figures and images for: Development of lacrimal gland organoids from iPSC derived multizonal ocular cells
Source: Front Cell Dev Biol. 2023 Jan 4;10:1058846. doi: 10.3389/fcell.2022.1058846 (PMC9846036; doi:10.3389/fcell.2022.1058846)

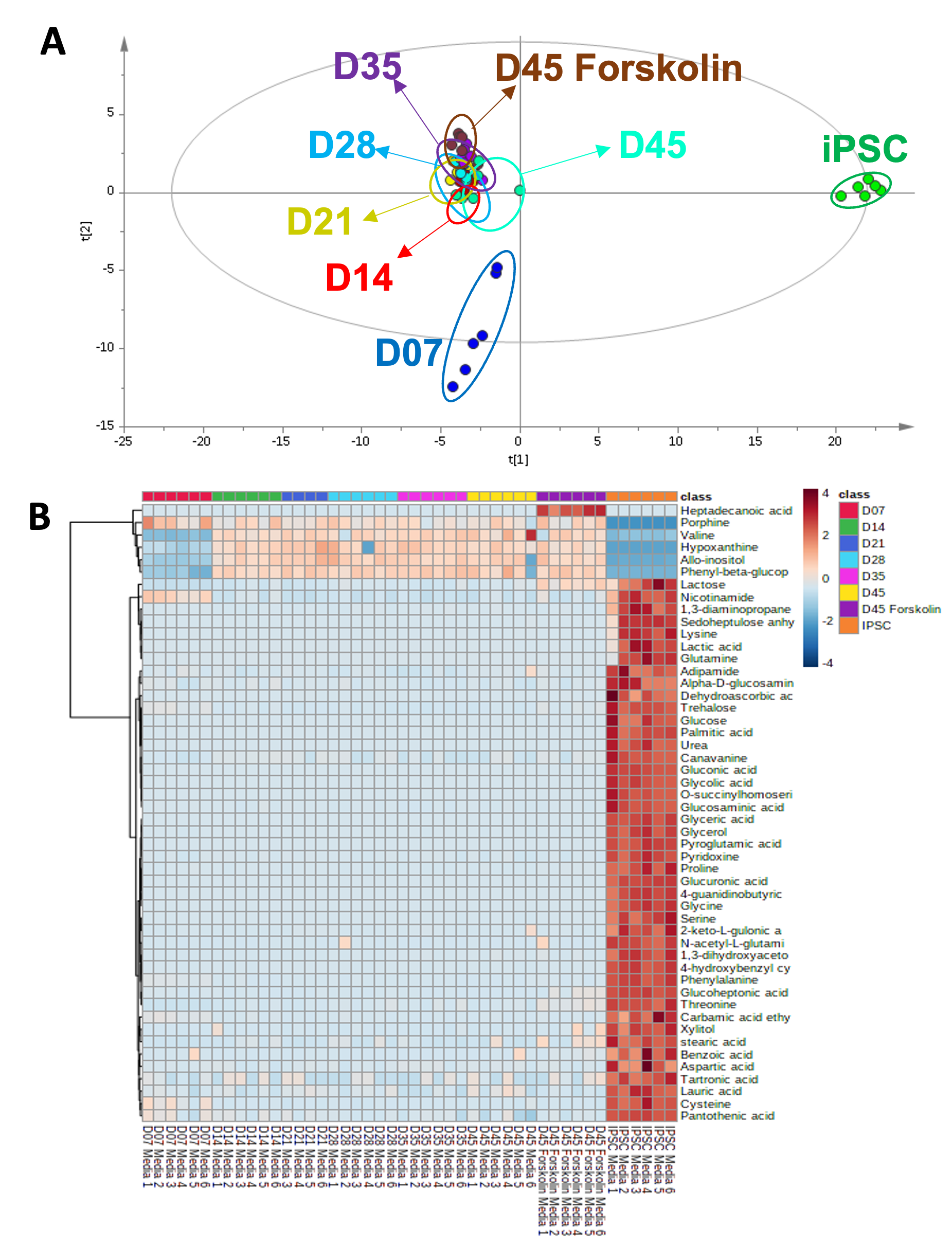

Supplement: Supplementary file 2 [file Image3.TIF]

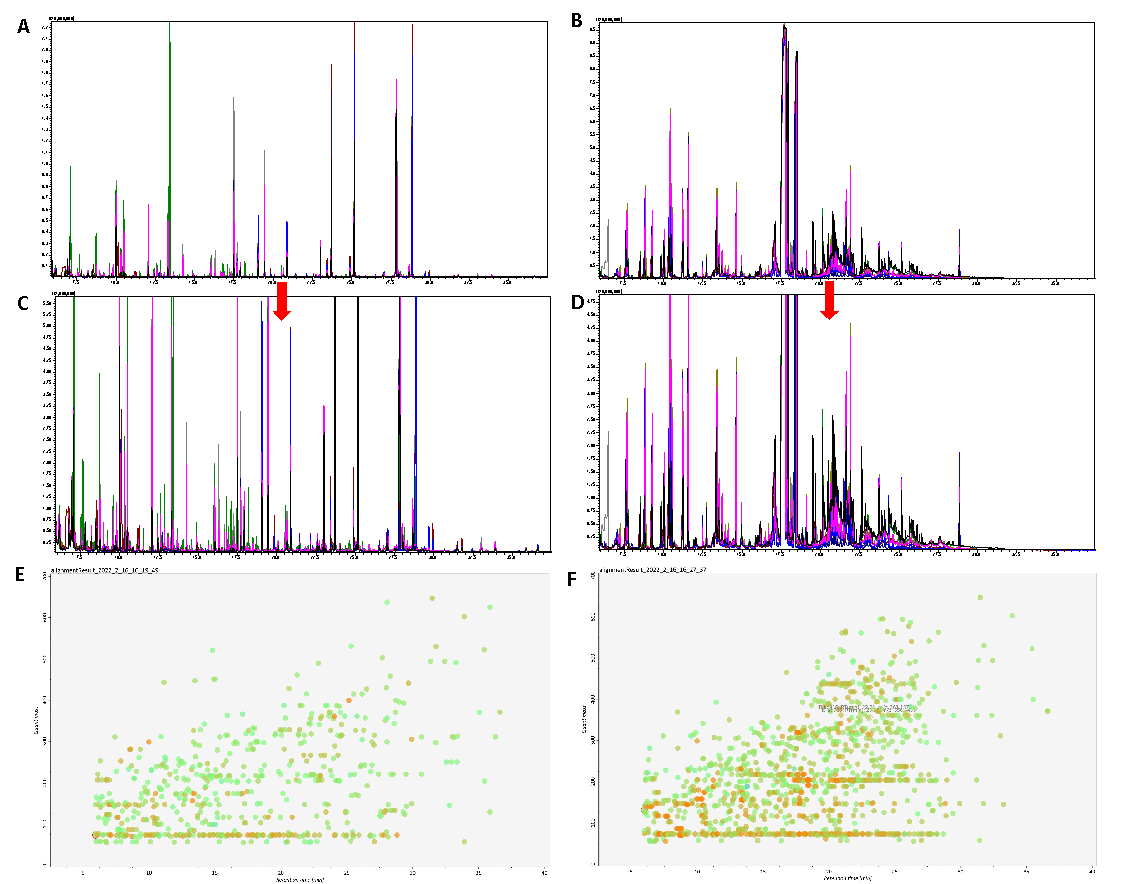

Supplement: Supplementary file 3 [file Image4.TIF]

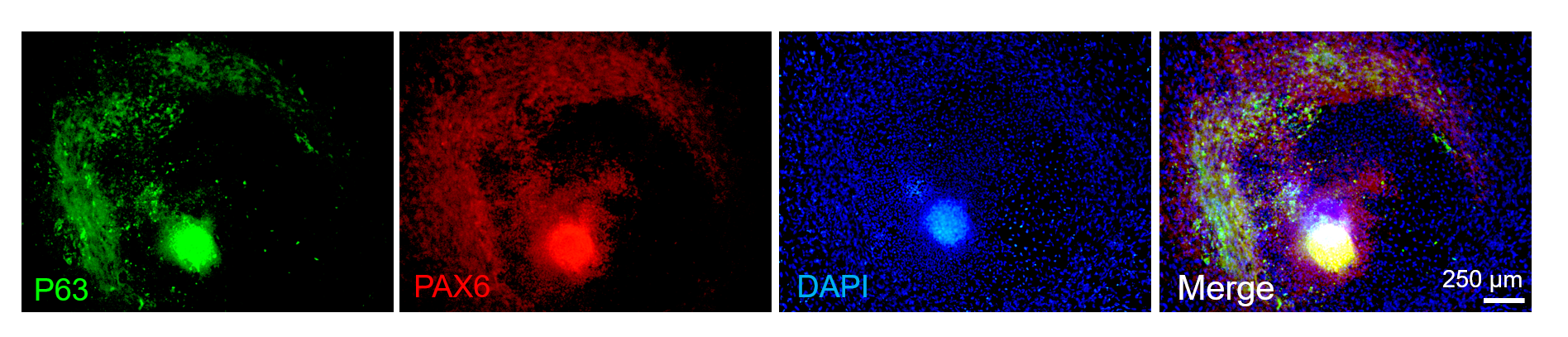

Supplement: Supplementary file 4 [file Image2.TIF]

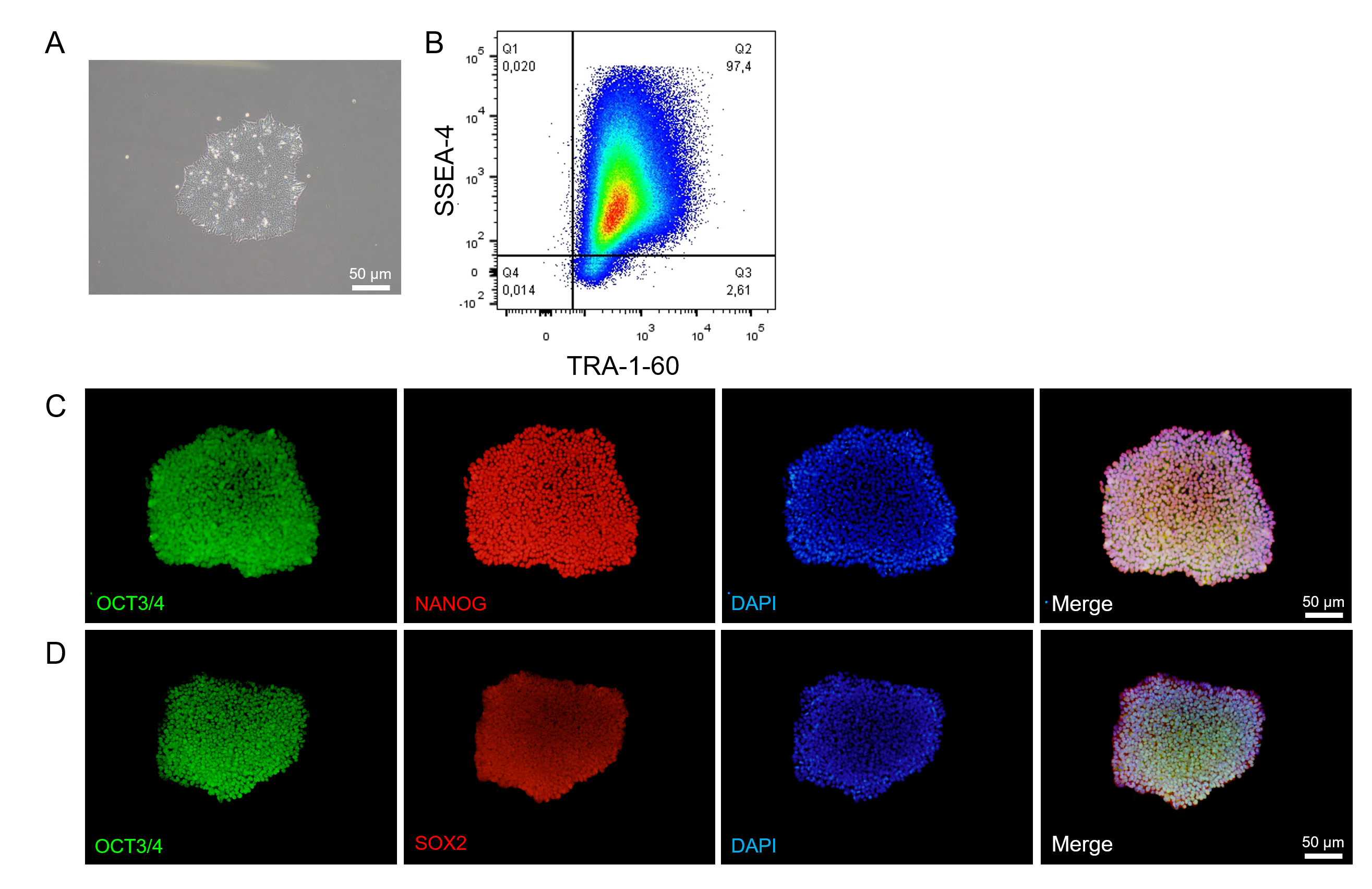

Supplement: Supplementary file 5 [file Image1.TIF]
