# Supplementary material for: Development of lacrimal gland organoids from iPSC derived multizonal ocular cells
Source: Front Cell Dev Biol. 2023 Jan 4;10:1058846. doi: 10.3389/fcell.2022.1058846 (PMC9846036; doi:10.3389/fcell.2022.1058846)
Supplement: Supplementary file 6 [file Table2.DOCX]

**Supplementary Table 2. Primer sequences for qPCR.**

| **Gene Name** | **Sequence (5’ -3’)** | **Gene Accession Number** |
| --- | --- | --- |
| ***GAPDH*** | ACCACAGTCCATGCCATCAC  TCCACCACCCTGTTGCTGTA | NM_002046 |
| ***PAX6*** | CAGAGCCCCATATTCGAGCC  CAAAGACACCACCGAGCTGA | NM_001604 |
| ***KRT13*** | ACGCCAAGATGATTGGTTTC  CGACCAGAGGCATTAGAGGT | NM_153490 |
| ***P63*** | GCAGTCGAGCACCGCCAAGT  TCAAAGCAGCGTCGGCCCAG | NM_003722 |
| ***FOXC1*** | CATTTTGGTCTAGGGTGGTTTC  TCTGATTGGCAGGGCAGAT | NM_001453 |
| ***LMX1B*** | GTGTGAACGGCAGCTACGC  TCATCCTCGCTCTTCACGG | NM_001174146 |
| ***LYZ*** | GCTGGAGACAGAAGCACTGAT  ATGCCTTGTGGATCACGGAC | NM_000239 |
| ***AQP5*** | GAACCCAGCCCGCTCTTTTG  AGTCCTCGTCAGGCTCATACG | NM_00165 |
| ***KRT15*** | ATCGCTACTTACCGCAGCC  CACCTGTCCATCCACTGACTC | NM_002275 |
| ***P75*** | CAGGACAAGCAGAACACCGT  GGTGTGGACCGTGTAATCCA | NM_002507 |
| ***FGF10*** | GAAATCGGAGTTGTTGCCGTC  TGCCACATACATTTGCCTCC | NM_004465 |
| ***SOX10*** | CCAGGCCCACTACAAGAGC  CTCTGTCTTCGGGGTGGTTG | NM_006941 |
| ***OTX1*** | AGACGCATCAGACCCTGAAGGACT  CTGCATACACGAGGTGTTGCTAGG | NM_001199770 |
| ***BMP7*** | TCCAAGACGCCCAAGAAC  ACAGCTCGTGCTTCTTACAGG | NM_001719 |
| ***LF*** | TTCGTTTGCCAAGTCGCCTC  ATTTTGTGGCCTCGGGTTGG | NM_002343 |
