# Supplementary material for: Development of lacrimal gland organoids from iPSC derived multizonal ocular cells
Source: Front Cell Dev Biol. 2023 Jan 4;10:1058846. doi: 10.3389/fcell.2022.1058846 (PMC9846036; doi:10.3389/fcell.2022.1058846)
Supplement: Supplementary file 7 [file Table3.DOCX]

**Supplementary Table 3. Antibodies used for flow cytometry.**

| **Target** | **Product number** |
| --- | --- |
| **Ep-CAM (CD326)-** **FITC** | Biolegend, 324204 |
| **P75 (CD271)-** **PE-Cy7** | Biolegend, 345110 |
| **TRA-1-60-R-** **AF488** | Biolegend, 330613 |
| **SSEA-4-** **AF647** | Biolegend, 330407 |
